# Decoding Early-Onset Aging After Cancer: Hallmarks, Biomarkers, and Future Directions for Childhood and Young Adult Survivorship

**DOI:** 10.3390/cancers18040644

**Published:** 2026-02-16

**Authors:** Jasper David Feldkamp, Nele Schmitt, Sanem Özayral, Mareike Frick

**Affiliations:** 1 Department of Hematology, Oncology and Cancer Immunology, Charité—Universitätsmedizin Berlin, Campus Charité Mitte, Charitéplatz 1, 10117 Berlin, Germany; jasper.feldkamp@charite.de; 2 Berlin Institute of Health at Charité—Universitätsmedizin Berlin, BIH Biomedical Innovation Academy, BIH, Charité Clinician Scientist Program, Charitéplatz 1, 10117 Berlin, Germany; 3 Department of Hematology, Oncology and Cancer Immunology, Charité—Universitätsmedizin Berlin, Campus Virchow Klinikum, Augustenburger Platz 1, 13353 Berlin, Germany; nele.schmitt@charite.de (N.S.); sanem.oezayral@charite.de (S.Ö.)

**Keywords:** cancer survivor, children, adolescents and young adults (CAYA), biomarker, methylation analyses, genomic profiling, inflammaging, cellular senescence, telomeres, mitochondria

## Abstract

Survival rates for children, adolescents, and young adults (CAYA) with cancer have markedly improved over recent decades, resulting in a rapidly growing population of long-term survivors. However, many of these individuals experience late and long-term treatment-related effects that resemble conditions typically associated with advanced age, including cardiovascular disease, endocrine dysfunction, neurocognitive impairment, and secondary malignancies. This clinical constellation has led to the concept of therapy-induced accelerated aging, suggesting that cancer treatments provoke biological changes that mirror, and may accelerate, physiological aging processes. In this review, we examine current evidence for aging-associated molecular hallmarks in CAYA cancer survivors, focusing on epigenetic alterations, genomic instability, chronic inflammation, cellular senescence, telomere attrition, and mitochondrial dysfunction. Epigenetic age acceleration is consistently observed across multiple survivor cohorts and correlates with treatment exposures, lifestyle factors, and chronic health conditions, positioning DNA methylation-based clocks as promising integrative biomarkers. Likewise, clonal hematopoiesis—reflecting persistent genomic stress—appears enriched in survivors, particularly decades after therapy, and may serve as an indicator of long-term cardiovascular and hematologic risk. Immune dysregulation, inflammaging, and senescence markers further underscore the systemic impact of cancer therapies on biological aging pathways. While telomere shortening and mitochondrial alterations also contribute to this phenotype, their standalone biomarker utility remains limited. Together, these hallmarks highlight the multifaceted nature of accelerated aging in CAYA survivors. Future work integrating multi-omics biomarkers with clinical phenotyping will be essential to identify high-risk individuals, guide targeted interventions, and advance personalized survivorship care.

## Simple Summary

More children, teenagers, and young adults survive cancer today than ever before, but many develop health problems years after their treatment has ended. These problems often look similar to conditions usually seen in much older adults, such as heart disease, hormone changes, memory difficulties, or second cancers. This has raised the important question of whether cancer treatment speeds up the body’s natural aging processes. In this review, we bring together current knowledge about biological changes that may explain why some survivors experience earlier or more severe long-term health issues. We look at different signs of aging inside the body, such as changes in DNA regulation, stress on blood-forming cells, inflammation, immune system weakness, and energy production in cells. By summarizing the latest research, we aim to show how these changes develop, how they may interact, and how they could help identify survivors at higher risk for future health problems. Understanding these biological aging processes may guide new ways to monitor health, prevent complications, and design treatments that protect long-term wellbeing in young cancer survivors.

## 1. Introduction

**Figure 1:**
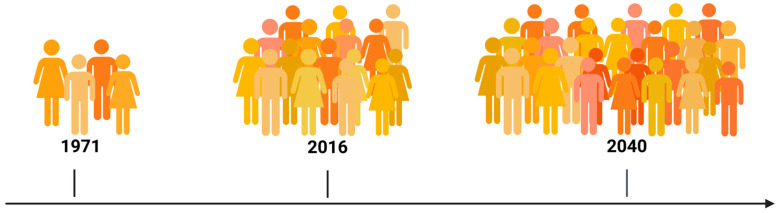
Increasing numbers of cancer survivors illustrate the dimensions and implications cancer survivorship care will have in the future. Data derived from [1]. One person symbolizes 1 million cancer survivors (all ages), data based on U.S. population. Created in BioRender. Frick, M. (2026) (https://BioRender.com/x5tyjuh. URL accessed on 20 January 2026).

Over the past decades, survival across many tumor types has substantially improved thanks to progress in cancer detection and cancer treatment. A significant increase in the number of cancer survivors is expected and we see rising numbers from 3.6 million (1971) to 15.5 million (2016). By 2040, this value is expected to be even higher, reaching 26.1 million survivors (data for U.S.) [1] (Figure 1). Nevertheless, with more patients surviving from cancer, the therapy they were exposed to often causes late and long-term effects (LLTEs). By definition, long-term effects begin during treatment and continue beyond end of treatment. Late effects, on the other hand, only appear after cancer treatment ended [1]. Children, adolescents and young adults (CAYA) are commonly defined as patients from 0 to 40 years and represent a large and growing group of cancer survivors worldwide. This results in a large population of long-term survivors with a high number of saved years of life; however, most CAYA survivors remain at risk for treatment-related late effects [2,3,4,5]. The risk of serious outcomes increases over time. Almost two thirds of childhood cancer survivors develop at least one chronic health condition and more than a fourth even suffer from severe or life-threatening disease and therapy sequelae [3,6]. In a comprehensive study, childhood cancer survivors were 3.3 times more likely than their siblings to suffer from a chronic illness [3]. Another study reports that childhood and AYA cancer survivors not only have a significantly higher risk of chronic diseases but also of increased long-term mortality. Specifically the standardized mortality risk for all-cause mortality was 5.9 in for AYA survivors and even 6.2 for children, when compared to the general population [4]. In summary, the late effects in AYA cancer survivors are extremely heterogeneous in manifestation and severity and can affect virtually every organ system.

Remarkably, we can observe clear parallels between treatment-associated sequelae and the aging human organism. Looking at the sum of LLTEs, we observe a clinical picture that closely resembles what we see in geriatric medicine [5]. To illustrate this in detail, various organ-specific LLTEs otherwise known from elderly populations will be briefly discussed in the following paragraph.

**Figure 2:**
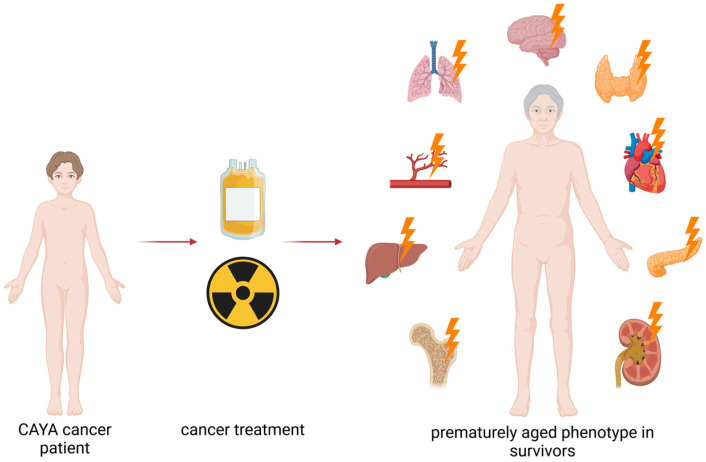
Clinical late and long-term effects after cancer therapy. Created in BioRender. Frick, M. (2026) (https://BioRender.com/x5tyjuh. URL accessed on 20 January 2026).

Endocrine complications are one of the most common side effects, including thyroid dysfunction, gonadal failure, infertility, early menopause and growth hormone deficiency [7,8,9]. Even at a relatively young age, changes in bone metabolism can contribute to osteoporosis [10]. Increased frequencies of cardiac events are also a serious late effect observed in CAYA survivors [1,11]. For childhood cancer survivors, their relative risk—compared to their healthy siblings—of suffering from congestive heart failure as a result of cancer and its treatment is estimated to be 15.0 [3]. Metabolic disorders such as insulin resistance, obesity and metabolic syndrome further intensify this cardiovascular risk [2,12]. Neurocognitive problems are equally relevant and often long-lasting. Many survivors describe fatigue, difficulties with attention and memory and slower cognitive processing [13]. Treatment-related secondary malignancies can be hematologic or solid, and are the most serious and fatal long-term consequences for cancer survivors [1,2,14] (Figure 2).

LLTEs can be present to varying degrees in individual survivors. However, if we look at the general clinical picture resulting from these conditions, it is characterized by a global, early, functional decline, closely resembling a phenotype associated with aging [5]. Normally, the above discussed conditions usually present with older age, but obviously appear much earlier in CAYA cancer survivors, often decades before expected under physiologic conditions. This observation has fostered the idea of cancer and therapy-induced accelerated aging [5], suggesting that cancer treatment can cause biological changes that are similar to those of the physiologic aging process. Clinically, this can be seen as a mismatch between chronological and perceived biological age [1,5].

However, not every survivor is equally affected. Known factors that have an impact on long-term outcomes are treatment intensity, genetic predisposition, comorbidities and lifestyle. For example, higher treatment intensity and younger age at diagnosis were associated with an increased risk of multiple organ dysfunction and functional loss [5]. However, even with the same treatment, the amount and degree of late effects can be very heterogeneous and risk factors as well as protective factors are yet incompletely understood. It is therefore of high interest to survivors, caregivers and researchers to better understand and characterize the biological background and underlying mechanisms that lead to these phenotypes. It therefore appears reasonable to analyze selected molecular features of aging in the context of cancer survivorship to guide a rational approach to molecular changes and subsequent biomarker detection and successful design of clinical intervention strategies.

## 2. Hallmarks of Aging and Their Association with Cancer Therapies

Aging is an inevitable and complex process. The twelve “hallmarks of aging”—systematically conceptualized by López-Otín in 2013 [15] and revised in 2023 [16]—are a widely accepted framework which describes aging as the consequence of specific cellular and molecular processes that lead to functional decline and increased susceptibility to disease over time. These twelve hallmarks are all connected with each other. According to the authors, aging is a “progressive loss of physiological integrity” that makes us more vulnerable to disease and death [15,16]. Notably, the authors also highlight that cancer and aging share a common basis in the accumulation of cellular damage [17] and additionally defined three criteria that each hallmark of aging should follow: it should be part of the normal aging process, its experimentally induced aggravation should accelerate aging and its reduction should slow aging [16].

**Figure 3:**
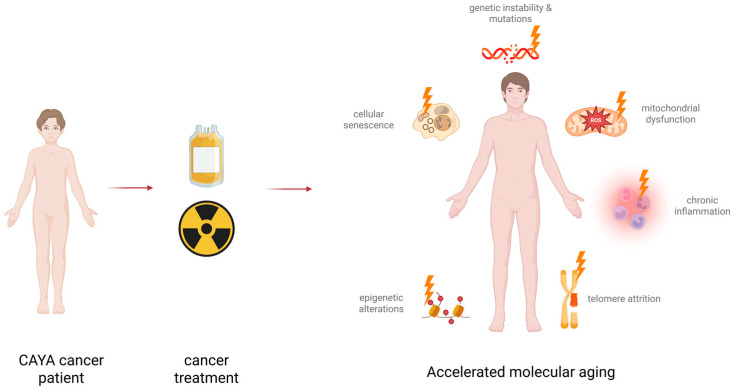
Selected molecular hallmarks of aging according to [16], as discussed in this review. These hallmarks provide a useful framework to systematically investigate molecular sequelae of cancer therapy. Created in BioRender. Frick, M. (2026) (https://BioRender.com/x5tyjuh. URL accessed on 20 January 2026).

In light of the obvious parallels between LLTEs observed in cancer survivors, the well-defined hallmarks of aging appear to be a useful concept for the evaluation of effects induced by cancer and its treatments. In recent years, relevant progress has been made in the systematic investigation of aging-associated molecular features in cancer survivors. In this review, we will present and discuss the current state of knowledge for the hallmarks most intensively studied. Specifically, we will focus on epigenetic alterations, genomic instability, chronic inflammation, cellular senescence, telomere attrition and mitochondrial function (Figure 3).

**Epigenetic alterations** are among the most informative molecular markers of aging. Changes in DNA methylation accumulate in a predictable manner across a lifespan, and “epigenetic clocks” were originally developed to predict chronological age. Deviations from this prediction have proven to correlate with age-related diseases and phenotypes [18]. Therefore, epigenetic age acceleration can be interpreted as an approximation to biological age. In addition to DNA methylation, age-associated epigenetic changes include histone modifications and chromatin restructuring, which influence gene expression and genome stability [19].

Early studies in survivors of childhood cancer employed first-generation epigenetic clocks (e.g., Weidner’s [20] and Horvath’s [21] clocks) and demonstrated significant epigenetic age acceleration both across immune cell subpopulations and in comparison with healthy siblings [20,21,22,23]. With increasing availability of DNA methylation array technology, larger cohort studies became feasible, coinciding with continued refinement of epigenetic clocks. A large number of observations were made in the childhood cancer survivor study, which compared individuals treated for cancer as children, adolescents and in part young adults (≥5 years post treatment; age at diagnosis range 0–23.6 years) to controls with no history of cancer [24]. Cancer survivors exhibited accelerated epigenetic age as measured by DNAm PhenoAge, a second-generation epigenetic clock with improved predictive power for disease risk and mortality [25]. Notably, epigenetic aging was particularly accelerated in individuals who had received chest radiotherapy, abdomen or pelvic radiotherapy, alkylating agents, glucocorticoids, or epipodophyllotoxins. Likewise and also noteworthy, unhealthy lifestyles were associated with accelerated epigenetic age, which, again correlated with a number of chronic health conditions such as hypertension, obesity, pulmonary problems and polyneuropathy [24].

Subsequent studies leveraging subsets of or extensions to this cohort corroborated and extended these findings. Plonski et al. demonstrated a higher annual change in epigenetic acceleration among children and adolescent cancer survivors compared to older survivors. In addition, the authors described an association between epigenetic age acceleration and early-onset obesity and a composite measure of burden of disease [26]. Similarly, Williams et al. reported epigenetic age acceleration among survivors with an increased deficit accumulation index, a composite marker of chronic health conditions and functional/psychosocial wellbeing [27]. Addressing racial and socioeconomic disparities, Chen et al. reported significantly increased epigenetic age acceleration among non-Hispanic Black cancer survivors and a comparable association for educational attainment [28]. Meng et al. were then able to show the mediating effect of epigenetic age acceleration on cardiometabolic risk factors and cardiovascular diseases among cancer survivors [29]. Within this cohort, epigenetic age acceleration as compared to healthy controls was also observed in further refined epigenetic clocks such as PCPhenoAge, PCGrimAge—preexisting clocks with improved reliability through principial component (PC) analysis—and DunedinPACE, a third-generation epigenetic clock designed to capture the pace of aging [30,31]. Williams et al. extended these findings with an association of accelerated epigenetic age and worse neurocognitive function. More specifically, accelerated age measures in the DunedinPACE and PCGrimAge were associated with worse performance on multiple measures of attention, processing speed, and executive functions. The authors hence conclude that the applied epigenetic clocks could be implemented for risk estimation for accelerated cognitive aging or be used as efficacy biomarker for neurocognitive interventions [32]. Studies with high relevance to the topic of comprehensive methylation analyses and cancer survivorship in CAYA patients are listed in Table 1.

This general association of epigenetic age acceleration in childhood, adolescent and young adult cancer survivors has been replicated in several smaller cohorts [33,34]. Notably, this observation was also made in a longitudinal study by Robinson et al., investigating samples before and after therapy. Using comprehensive analysis of the generated methylation data, the authors identified several differentially methylated regions that could potentially serve as biomarkers for assessing toxicity of cancer treatments and for predicting long-term health outcomes in childhood cancer survivors [35].

Hence, comprehensive methylation analyses bear an enormous potential to determine and even quantify a plethora of biological processes associated with or mimicking aging processes. Respective analyses are therefore of high interest to survivors and researchers. Though implementation of epigenetic analyses will most probably not be part of regular survivorship care in the near future, respective analyses will help to better understand long-term systemic responses and side effects of cancer therapies and also have a high potential to serve as biomarkers in prospective intervention studies.

**Table 1:** Summary of current literature concerning epigenetic aging in CAYA cancer survivors.

Author	Year	Title	Observations	Platform	Clocks	Comparison
Daniel S. et al. [22]	2018	T cell epigenetic remodeling and accelerated epigenetic aging are linked to long-term immune alterations in childhood cancer survivors.	44	CpG-Site Specific Pyrosequencing	Weidner’s Clock	In between T-Lymphocytes
Wang J. et al. [23]	2019	DNA methylation patterns of adult survivors of adolescent/young adult Hodgkin lymphoma compared to their unaffected monozygotic twin.	9	Human-Methylation27	Horvath’s Clock	Twin Study
Qin N. et al. [24] *	2021	Epigenetic Age Acceleration and Chronic Health Conditions Among Adult Survivors of Childhood Cancer.	1563	EPICv1	DNAmPhenoAge	Healthy Controls
Robinson N. et al. [35]	2022	Anti-cancer therapy is associated with long-term epigenomic changes in childhood cancer survivors.	32	EPICv1	Skin&Blood, (GrimAge)	Healthy Controls
Plonski N.M. et al. [26] *	2023	Epigenetic Age in Peripheral Blood Among Children, Adolescent, and Adult Survivors of Childhood Cancer.	2846	EPICv1	DNAmPhenoAge, (Horvath’s Clock), (Hannum’s Clock), (GrimAge)	Age Groups
Williams A.M. et al. [27] *	2023	Deficit Accumulation Index and Biological Markers of Aging in Survivors of Childhood Cancer.	2101	EPICv1	DNAmPhenoAge	DAI Groups
Gehle S.C. et al. [33]	2023	Accelerated epigenetic aging and myopenia in young adult cancer survivors.	58	EPICv1	DNAmPhenoAge, GrimAge	Healthy Controls
Harris R.D. et al. [34]	2023	Epigenetic age acceleration among survivors of pediatric medulloblastoma and primitive neuroectodermal tumor.	83	Methylation450k	Horvath’s Clock	No Controls
Chen C. et al. [28] *	2024	Race and Ethnicity, Socioeconomic Factors, and Epigenetic Age Acceleration in Survivors of Childhood Cancer.	1706	EPICv1	DNAmPhenoAge	Race/Ethnic/SDOH Groups
Meng X. et al. [29] *	2025	Epigenetic Age Acceleration Mediates Treatment Effects on Cardiometabolic and Cardiovascular Risk in Childhood Cancer Survivors.	2939	EPICv1	DunedinPACE, PCPhenoAge, GrimAge2	Treatment Modalities/CMRFs
Williams A.M. et al. [32] *	2025	Epigenetic age acceleration, telomere length, and neurocognitive function in long-term survivors of childhood cancer.	1413	EPICv1	PCPhenoAge, PCGrimAge, DunedinPACE, (Horvath’s Clock), Hannum	Healthy Controls

* Marks studies from the St. Jude’s childhood cancer survivor cohort. Epigenetic clocks that did not yield significant results are shown in brackets. DAI: deficit accumulation index. SDOH: social determinants of health. CMRFS: cardiometabolic risk factors.

**Genomic instability** is another central hallmark of aging. DNA damage accumulates naturally with age. It can be caused by external factors such as radiation, chemicals, or viral integrations, as well as by internal threats such as replication errors, reactive oxygen species, and spontaneous hydrolytic reactions. Antineoplastic therapies may further contribute to long-lasting genomic instability in healthy tissues [36]. Clonal hematopoiesis (CH) clonal hematopoiesis is defined as a disproportionate proliferation of hematopoietic stem cells driven by somatic, hence acquired, mutations in genes typically associated with hematologic malignancies. Also referred to as age-related clonal hematopoiesis (ARCH), it is associated with an increased risk of age-related diseases such as cardiovascular diseases and hematopoietic malignancies [37,38] and can therefore be considered a suitable biomarker of aging linked to genomic instability. Prevalence of clonal hematopoiesis is strongly dependent on the method used for detection and the variant allele frequency (VAF) cutoff applied; however, clones with a VAF ≥ 2% (then meeting the definition for clonal hematopoiesis of indeterminate potential = CHIP) are rare in individuals of 40 years or younger [39].

While growing evidence suggests a higher prevalence and a distinct mutational spectrum of clonal hematopoiesis, skewed toward mutations in DNA damage repair genes in adult cancer survivors [14,40], studies in young cancer survivors remain limited. The earliest study addressing this question was unable to confirm the notion of more abundant clonal hematopoiesis in 84 young cancer survivors compared to healthy controls [41]. This observation was later contradicted by data from the St. Jude’s Childhood Cancer survivor Study comprising 2860 survivors from pediatric cancer, which demonstrated significantly higher rates of clonal hematopoiesis compared with healthy controls. This effect was most pronounced in survivors treated with alkylating agents, radiation, and bleomycin. Mutations in *TP53* and *STAT3* were considered to be therapy-related. Longitudinal analyses revealed that clones with mutations in age-related CH genes (*DNMT3A*, *TET2*, *ASXL1*) tended to increase in VAF over time, while VAF of treatment-related clones stayed relatively stable over time [42]. Notably, to address the contradictory findings of the two studies, the median follow-up time since therapy was highly different between the two cohorts. While median follow-up in the study by Collord et al. [41] was around 6 years, it was more than 23 years in the study by Kohei et al. [42]. As we know that clone size is subject to dynamic changes over years and decades, this might at least in part explain the different findings. An association between cancer therapy and clonal hematopoiesis in young and childhood cancer survivors was further confirmed in a smaller cohort of 100 individuals. As expected, *DNMT3A*, *TET2*, *ASXL1*, *PPM1D*, *TP53* and *CHEK2* were most frequently mutated. In this study, an increased frequency of clonal hematopoiesis in cancer survivors was shown when compared to cancer controls who had been newly diagnosed with cancer and had not received treatment at the time of sampling. This indicates that mutations and clonal expansion are at least in part caused by cancer therapy, leading to replicative stress and evolutionary pressure on the hematopoietic system. Interestingly, individuals in this cohort also included adolescents and young adults, as the median age of treated cancer survivors was 19 years [43]. The authors conclude that clonal hematopoiesis could potentially drive the long-term high-burden of premature multimorbidity and secondary myeloid neoplasms [43]. A study investigating the frequency of clonal hematopoiesis in 878 young women aged 40 years or younger treated for breast cancer detected somatic driver mutations in 28.7% of study participants, but only 2.7% had a VAF of >2%, meeting the criteria for CHIP. In this cohort, during a median follow-up time of nine years, no elevated risk for adverse outcomes could be detected [44].

In summary, various studies could show that clonal hematopoiesis is enriched in survivors of childhood and AYA cancers when compared to healthy or untreated controls. These findings emphasize the role of cancer therapy as an evolutionary bottleneck, favoring the outgrowth of mutated clones. However, in the CAYA population, clone size rarely exceeds the 2% VAF threshold (criterion for meeting the definition of CHIP) and clinical implications—for example, therapy-associated myeloid malignancies and increased frequency of cardiovascular events—need to be thoroughly evaluated in future studies. Additionally, given its well-described impact in the elderly population with increased risk for cardiovascular and other inflammatory diseases, detection of clonal hematopoiesis in CAYA cancer survivors appears as a promising and robust biomarker for risk estimation that is worthy of future in-depth evaluation.

In contrast to clonal hematopoiesis, cancer predisposition syndromes are inherited conditions. However, as CH, they are often associated with genomic instability, increasing the risk for subsequent cancers. Across sequencing studies, approximately 8–16% of children with cancer have cancer-predisposing variants [45,46,47]. In line with these findings, it has been shown that long-term cancer survivors often carry pathogenic/likely pathogenic variants [48]. Identifying these variants is important as the carriers are at an increased risk of developing subsequent neoplasms [49]. In patients with cancer predisposition syndromes, a significant aspect in survivorship care is elucidating the cancer risks related to the syndrome. For example, Lynch syndrome is caused by DNA mismatch repair (MMR) genes such as *MLH1*, *MSH2*, *MSH6*, and *PMS2*, a condition that increases the risk for various cancers, some of which are not typically seen in children [50]. Another example is Li-Fraumeni syndrome, which is caused by germline *TP53* variant. Here, we see high rates of not only early-onset but also multiple primary malignancies [51]. Beyond MMR and *TP53*, other genes such as *BRCA1/2*, *PALB2*, *CDKN2A*, *PTEN*, *STK11*, *NF1*, *DICER1* were reported to be associated with multiple primary cancers [48]. Overall, characterization of germline cancer predisposition in cancer survivors can refine risk-stratified surveillance and aid in developing preventive strategies.

**Chronic Inflammation/Cellular Senescence:** Immune dysregulation and alteration of the immune system are key short-term and long-term side effects caused by cancer and its therapies. Cancer therapies can induce broad and lasting disruptions of systemic immunity that extend well beyond the tumor microenvironment. In the weeks and months before, during and following cancer therapy, tumor growth skews bone marrow hematopoiesis toward immature neutrophils and monocytes, expanding myeloid-derived suppressor cells that circulate and promote immunosuppression [52,53,54,55,56]. Peripheral dendritic cells decline in number and function, impairing antigen presentation and CD8^+^ T cell priming [57,58]. Circulating T cells show reduced receptor diversity, diminished cytokine production, and increased regulatory T cell expansion, while regulatory B cells and dysfunctional natural killer cells accumulate [59,60,61,62,63]. These changes collectively shift the immune system toward an anti-inflammatory, tumor-permissive state. Conventional therapies reinforce or reshape these alterations. Despite well-known side effects on the myeloid compartment, chemotherapy and radiation can cause long-term lymphopenia and persistent biases in memory T cell subsets [64]. Importantly, these systemic perturbations also blunt responses to unrelated infections or vaccinations, reflecting compromised dendritic cell activation and CD8^+^ T cell differentiation [65,66]. Together, these deep immunological changes following cancer and its treatments can be summarized as “immunological scars”. Of note, many of these changes have overlapping features with immunological and inflammatory changes associated with aging and are hence termed “inflammaging” [67]. The concept of “immunological scarring” and “inflammaging” in the context of cancer survivors receives increasing attention, though due to the complexity of the topic, comprehensive analyses remain challenging.

A recent in-depth immunological analysis of long-term survivors of diffuse large b cell lymphoma (DLBCL) could prove that “immunological scars” persist years after therapy [68]. The study was performed in a population of adult cancer patients, including but not restricted to young adults. The “immunological scars” described in the publication were characterized by high IL6, high myeloid-derived suppressor cells, and low immature T cell counts and impaired adaptive immunity. Moreover, the authors could show that the same immunological alterations already exist at first diagnosis, fostering speculations that preexisting alterations support the development of cancer. Notably, these alterations could likewise be interpreted as markers of aging-associated inflammation (“inflammaging”) [69]. Interestingly, blood from AML, CLL, and breast cancer patients likewise showed sustained immune alterations years after therapy, with each malignancy having its own, unique immunological imprint [68]. Similar data support immunological scarring in long-term survivors of multiple myeloma [70]; however, this is a disease that usually appears in the elderly. In summary, sustained immunological changes are measurable in long-term survivors of both hematologic and solid cancers.

Cellular senescence and chronic inflammation are deeply interconnected processes that drive physiological aging. Senescent cells, which enter a permanent state of growth arrest due to factors like telomere shortening [71], accumulate more rapidly in older individuals. These cells secrete senescence-associated secretory phenotype (SASP) factors, which can propagate senescence in neighboring cells [16] and contribute to a pro-inflammatory environment. While SASPs play a role in tissue repair and immune activation [72], their persistent presence fosters chronic inflammation, again contributing to inflammaging [73,74]. In immune cells this can induce a weakened immune response or disbalance among immune cells (immunosenescence or aging-related immune phenotype, ARIP). Aging processes stemming from senescence are at least codependent with aging resulting from inflammation and can be induced by many factors including cancer therapies [75,76]. Inflammaging is marked by elevated levels of pro-inflammatory cytokines such as IL1β, IL6, IL10, and TNFα, which are significantly higher in older populations and have been linked to cardiovascular diseases, cognitive decline, diabetes mellitus, and chronic kidney disease [77]. Likewise, increased SAPS and immunosenescence correlate with mortality and are established biomarkers of aging. Senescent cells show characteristic changes on morphological level and increased activity of senescence-associated β-galactosidase (SA-β-Gal) [78]. At the molecular level, possible indicators of cellular senescence include DNA damage response, secretion of inflammatory cytokines and chemokines, involvement of cyclin kinase dependent inhibitors (CDKs) and production of reactive oxygen species (ROS) [75,78].

Importantly, senescence can be triggered by DNA damage, infection, oxidative stress, and cancer therapies, leading to both protective and harmful effects [79,80]. Regarding the effect of these stressors on the immune system, immunosenescence weakens immunity and can cause chronic damage. Specifically, this can lead to a skew of myloid cells over lymphoid cells [81], a phenomenon also seen as an effect of cancer therapies [65]. Furthermore, at the T cell level, replicative senescent T cells are mainly terminal effector (TE) or effector memory (EM) cells, with increased expression of CDK inhibitors like p16 and p21, which block cell cycle progression [82]. Expression of p16INK4a has turned out to be a promising and practical marker in the measurement of senescence in general and has been implemented in cancer survivors. In a cohort of 60 children, adolescent and young adult cancer survivors, expression of p16INK4a was higher among survivors than among healthy controls and increased from pretherapeutic samples to posttherapeutic samples in samples taken longitudinally. Moreover, expression levels of p16INK4a correlated with clinically determined frailty status [83]. Another study in survivors of breast cancer could likewise show that p16INK4a expression was associated with higher levels of frailty [84]. Due to these correlations, p16INK4a expression levels are one of several primary outcome measures in a clinical intervention trial testing various senolytics in cancer survivors (NCT04733534). Likewise, IL6 levels correlated with frailty status in various studies; however, these studies mostly investigated older adults [85].

Together, immunological changes, chronic inflammation and cellular senescence following cancer therapies are complex and multifactor processes. However, relatively simple measurements such as analysis of immune cell subpopulations, IL6 levels or p16INK4a expression emerge to be useful tools to gain first insights into these processes and are evaluated as therapeutic markers in pharmacologic intervention studies.

**Telomere attrition** is a mechanism through which cancer therapy may accelerate biological aging. Treatments that enforce high proliferative stress on hematopoietic cells can lead to shortened leukocyte telomeres, a process also observed during normal aging [86]. Telomere shortening can induce cellular senescence, an aging hallmark described above. A series of studies have described shortened telomeres in survivors of childhood cancer. In a large study with almost 2500 cancer survivors, Song et al. could show leukocyte telomeres were significantly shorter than in healthy controls. Moreover, chest or abdominal irradiation, glucocorticoid, and vincristine chemotherapies could be identified as risk factors for telomere shortening, while a healthy lifestyle seemed to have protective effects [87]. Interestingly, leukocyte telomere length does not seem to have an impact on neurocognitive function [32].

While shortening of telomeres is one of the oldest established measures of aging, its use as a biomarker is comparably low, particularly in light of the versatile information that can be derived from other markers. Notably, telomere length can be reliably derived from comprehensive methylation data using regression models [88].

**Mitochondrial function** is key to bioenergetics in all cells and is therefore central to many cellular processes. However, comparably little is known about altered mitochondrial function during or after cancer therapy. In a study investigating the antioxidant defenses and oxidative damage in mononuclear cells from childhood cancer survivors, antioxidant responses were impaired and oxidative stress increased. This ultimately led to a higher production of reactive oxygen species, similar to metabolic processes known from older individuals [89]. In another more clinical study, an association between the copy number of mitochondrial DNA and sarcopenia among childhood cancer survivors could be described [90], while on the contrary, increased mitochondrial DNA copy numbers were associated with decreased odds of abnormal cardiac function in childhood cancer survivors [91]. Notably, almost all studies use peripheral blood for analysis of mitochondria. However, a reduced number or altered function of mitochondria in specific tissues is well conceivable, but analyses of most other tissues remain challenging due to interventional risks when obtaining the tissues (e.g., cardiomyocytes, nerval tissues, etc.).

## 3. Discussion

The evidence summarized in this review supports the concept that cancer and its treatment can induce long-lasting molecular changes that closely resemble—and potentially accelerate—physiological aging in (CAYA) cancer survivors. Across multiple hallmarks of aging, survivors exhibit biological features that are typically associated with much older individuals, reinforcing the notion of therapy-induced accelerated aging as a unifying framework for understanding late and long-term effects. Importantly, these changes are neither uniform nor inevitable, highlighting the need for biologically informed risk stratification beyond conventional clinical predictors.

Among the investigated hallmarks, epigenetic alterations currently represent the most mature and quantitatively robust biomarker of accelerated aging in cancer survivorship. Multiple independent cohorts (summarized in Table 1) demonstrate consistent epigenetic age acceleration in CAYA survivors, with strong associations to treatment factors, lifestyle factors, and chronic health conditions. Second- and third-generation epigenetic clocks, particularly those designed to capture disease risk and pace of aging, appear especially promising for longitudinal monitoring and for use as surrogate endpoints in intervention trials. Their ability to integrate complex biological information into a single metric may allow early identification of survivors at increased risk for future health problems related to cancer treatment.

Clonal hematopoiesis emerges as a complementary biomarker reflecting therapy-induced genomic instability and altered hematopoietic stem cell fitness. While clone sizes in CAYA survivors often remain below classical CHIP thresholds, the enrichment of therapy-related mutations and their persistence decades after treatment underscore the long-term evolutionary consequences of genotoxic stress. Importantly, clonal hematopoiesis may represent both a marker and a mediator of downstream inflammatory and cardiovascular phenotypes. Future studies will need to clarify which clone characteristics—such as gene affected, variant allele frequency, or clonal dynamics—carry meaningful prognostic value in young survivors.

Chronic inflammation and cellular senescence provide a mechanistic link between multiple hallmarks of aging. The concept of “immunological scarring” integrates persistent immune dysregulation, inflammaging, and senescence-associated secretory phenotypes into a coherent model of long-term systemic vulnerability. Biomarkers such as IL6 levels, immune cell subset distributions, and p16INK4a expression are attractive due to their biological interpretability and clinical feasibility. Their inclusion as outcome measures in ongoing senolytic and anti-inflammatory intervention trials highlights their translational relevance.

Telomere attrition and mitochondrial dysfunction further contribute to the aging phenotype, although their standalone utility as clinical biomarkers appears more limited. Nonetheless, their integration into multi-parameter biomarker panels may improve biological resolution, particularly when derived indirectly from comprehensive methylation or multi-omics datasets.

It is important to note that not all LLTEs can be directly linked to one of the aging hallmarks by López-Otín and that additional observations should be considered. One example is the dysfunction of the autonomous nervous system (ANS) and cardiac autonomic dysfunction (CAD) respectively, in CAYA cancer survivors. For example, one third of survivors of childhood acute lymphoblastic leukemia suffer from this condition, which is associated with lower mean heart rate recovery and lower peak exercise tolerance [92], which are well-established features of aging. Therefore, on an organismal level, aging-associated features can again be observed and quantified, though one must hypothesize that this clinical condition is caused by a variety of molecular processes.

Notably, in terms of biomarkers, promising approaches already exist to monitor therapeutic toxicity during and after cancer therapy. In female cancer patients, premature ovarian failure is frequently observed among cancer survivors [93]. In part, ovarian function can be monitored through estimation of the Anti-Müllerian hormone (AMH) [94]. This is of particular interest in female CAYA cancer survivors especially concerning reproductive functions. Multiple studies could show that AMH can be used as a surrogate biomarker for the estimation of premature ovarian failure after anti-cancer therapy [95]. Notably, AMH levels can also be used to predict premature ovarian failure before and directly after the end of chemotherapy [96,97].

Another biomarker-based approach to monitor and prevent toxicity is the implementation of pharmacogenomic testing. Here, treatments and dosings are adapted to genetic variants known to have an impact on the toxicity of anti-cancer drugs. An established and well-known example of this approach is the testing of dihydropyrimidine dehydrogenase (DPD) variants before the application of 5-fluorouracil (5-FU) to avoid toxicity with maximum efficacy [98].

## 4. Conclusions

Looking forward, the greatest challenge and opportunity lie in integrating these diverse biomarkers into coherent, longitudinal models of survivorship care. Rather than relying on single markers, future strategies will likely combine epigenetic aging metrics, clonal hematopoiesis profiling, inflammatory signatures, and clinical phenotypes to define individualized trajectories of biological aging. Such approaches could enable early preventive interventions, guide survivorship surveillance, and ultimately shift care from reactive management of late effects toward proactive preservation of long-term health in CAYA cancer survivors.

## Data Availability

No new data were created or analyzed in this study.
